# Characterization of the complete chloroplast genome of *Poa pratensis* L. cv. Qinghai (Gramineae)

**DOI:** 10.1080/23802359.2019.1710276

**Published:** 2020-01-14

**Authors:** Linna Wei, Chunping Zhang, Quanmin Dong, Yang Yu, Xiaoxia Yang

**Affiliations:** State Key Laboratory of Plateau Ecology and Agriculture in the Three River Head Waters Region, Qinghai Academy of Animal and Veterinary Science, Qinghai University, Xining, Qinghai, China

**Keywords:** *Poa pratensis* L. cv. Qinghai, complete chloroplast genome, phylogenetic analysis

## Abstract

This study provides a chloroplast genome of *Poa pratensis* L. cv. Qinghai. The complete genome is 135,606** **bp in length with a G** **+** **C content of 38.28%, which contains 32 tRNA genes, and 8 rRNA genes. The phylogenetic analysis indicated that *P. pratensis* L. cv. Qinghai is closely related to *Festuca arundinacea cultivar KY-31*. These results contribute to explore the genetic evidence for adaptation to the Qinghai-Tibet Plateau.

The *Poa pratensisi* L. cv. Qinghai used in this study was the common and dominant species native to the QingHai-Tibet Plateau. Seeds were collected from Qinghai Province, Maqin county (N 34°47′, E 100°22′). The *P. pratensis* L. cv. Qinghai is an excellent species that has been selected and cultivated by our research group for a long time. Seeds were stored in cloth bags at 4 °C for 6 months to break the dormancy, following which, they were cleaned and air-dried. The *P. pratensis* L. cv. Qinghai seeds are stored in the Key Laboratory of Superior Forage Germplasm in the Qinghai-Tibetan Plateau, the specimen Accession number is ‘QC13’.

Kentucky bluegrass (*P. pratensis* L.), belongs to the genus *Poa*, in the family of Gramineae, which is a perennial grass species with a facultative apomictic cool-season widely used for forage and turf (Huff and Bara 1993; Huff 2003, 2010; Honig et al. 2012). In recent years, some new lines were bred to adapt to the local climate in China, such as *P. pratensis* L. cv. Daqingshan (Mh et al. 2000), *P. pratensis* L. cv. Huhe (He et al. 2009), *P. pratensis* L. cv. Huqing (Yuan et al. 2014). The Qinghai-Tibet Plateau is the world’s largest plateau, which is located in western China, with an average elevation of over 3000** **m above sea level. Drought and cold are the characteristics of the plateau. Wang et al. (2010) bred a new line for tolerance to low temperature and called it *P. pratensis* L. cv. Qinghai. In the past few years, some researches focused on the physiological mechanisms to drought tolerance and resistance and the molecular biological mechanisms, but there was lack of the genomic information of the *P. pratensis* L. Therefore, establishing a complete chloroplast genome of *P. pratensis* L. will contribute to explore the genetic evidence for adaptation to the Qinghai-Tibet Plateau.

In this study, *P. pratensis* L. cv. Qinghai seedlings were collected from Guinan county, Qinghai Province (35°28′13″N, 100°45′41″E). Total genomic DNA was extracted from the fresh and young leaves. DNA was used to construct a library for sequencing with the Illumina NovaSeq platform (Illumina, San Diego, CA, USA). NovoPlasty was used to assemble the complete chloroplast genome sequence (Dierckxsens et al. 2016). The complete chloroplast sequence of *P. pratensis* L. cv. Qinghai was deposited in the GenBank database with accession number (MN551182).

The complete chloroplast genome sequence of *P. pratensis* L. cv. Qinghai was 135,606** **bp in size, with a large single-copy (LSC) region of 85,152** **bp, and a small single-copy (SSC) region of 12,759** **bp. The overall G** **+** **C content of the chloroplast genome is 38.28%. The chloroplast genome contained 79 genes, including 8 rRNA genes and 32 tRNA genes.

We constructed a phylogenetic tree by using MEGA X (Kumar et al. 2018). The results showed that *P. pratensis* L. cv. Qinghai was closely related with *Festuca arundinacea cultivar KY-31*（Figure 1). The chloroplast genome of *P. pratensis* L.cv. Qinghai and phylogenetic tree analysis provided more valuable genomic information to explore inheritance and evolution.

**Figure 1.**
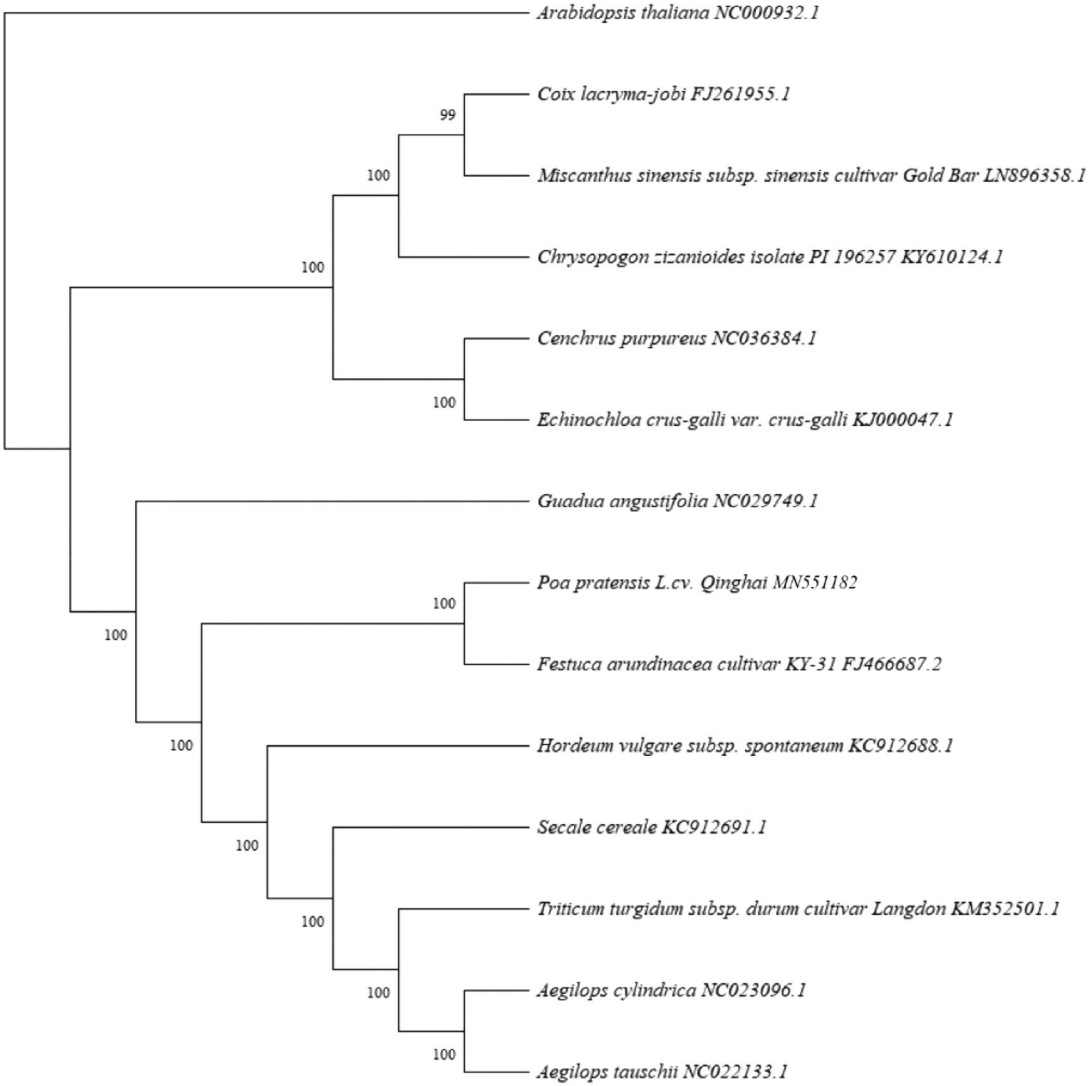
Maximum-likelihood phylogenetic tree based on 14 selected Gramineae complete chloroplast genome sequences.
